# Regeneration and Musculature in Halved *Cassiopea xamachana* Ephyrae

**DOI:** 10.1093/iob/obaf030

**Published:** 2025-07-26

**Authors:** K M Muffett, M Mammone, J Puckett, G Martino, Aditi, M P Miglietta

**Affiliations:** Department of Molecular and Cell Biology, University of California Merced, Merced, CA, USA; Department of Marine Biology, Texas A&M University Galveston, Galveston, TX 77554, USA; Department of Marine Biology, Texas A&M University Galveston, Galveston, TX 77554, USA; Department of Marine Biology, Texas A&M University Galveston, Galveston, TX 77554, USA; Department of Microbiology and Immunology, University of Texas Medical Branch, Galveston, TX 77555, USA; Department of Marine Biology, Texas A&M University Galveston, Galveston, TX 77554, USA

## Abstract

Adult *Cassiopea* medusae and their polyps have been known to regenerate tissue in uncontrolled and controlled conditions; however, the regeneration capabilities of *Cassiopea xamachana* ephyrae are largely unexplored. Here, we detail the development and regeneration of ephyrae under known laboratory conditions. Ephyrae were cut in two and then followed as they regenerated back to complete individuals. We visually document all the developmental stages of the medusa leading up to the trauma and the complete regeneration process of the two halves. We show how ephyrae of *C. xamachana*, when cut in halves, undergo both regeneration and re-symmetrization, generating, in about 2 weeks, two functional smaller ephyrae with fewer rhopalia and normal behavior. We also show that regeneration is slower in older ephyra.

## Italian

Le meduse adulte di *Cassiopea* e i loro polipi rigenerano i tessuti in condizioni controllate e in natura; tuttavia, le capacitã di rigenerazione delle efire di *Cassiopea xamachana* sono in gran parte inesplorate. Questo aricolo descrive in dettaglio lo sviluppo e la rigenerazione delle efire in condizioni di laboratorio. Le efire sono state sezionate in due e poi seguite mentre si rigeneravano fino a diventare individui completi. Documentiamo visivamente tutte le fasi di sviluppo della medusa dal trauma e il processo di rigenerazione completo delle due metã. Mostriamo come le efire di *C. xamachana*, se sezionate a metã, subiscano sia rigenerazione che risimmetrizzazione, generando, in circa due settimane, due efire piú piccole e funzionali con meno ropali e un comportamento normale. Inoltre dimostriamo che la rigenerazione é leggermente piú lenta nelle efire piú vecchie.

## Introduction

Marked changes in regenerative capacity exist across phylogeny and throughout ontogeny, with many organisms experiencing declining regenerative capacities over their lifespan (Yun 2015; Zattara and Bely 2016). Metazoan species exhibit a diverse range of lifespans, and the association between aging and regeneration potential has been shown in most organisms studied so far. Although most of these studies focus on vertebrates (Sousounis et al. 2014) or typical model systems, ontogenetic changes in regenerative capabilities have also been shown in some invertebrates (Jeffery et al. 2008). While variation in regeneration during development remains largely understudied in most invertebrate nonmodel systems, defining aging and senescence continues to be of interest in some invertebrate taxa (Schaible et al. 2015; Sahu et al. 2017). Scyphozoan jellyfish of the phylum Cnidaria exhibit a clear ontogeny that goes from planula, to polyp, to ephyra, to adult, to senescent medusa, and thus can be used to test how, and if, regenerative capabilities change during development (Helm 2018).

Regeneration in cnidarians is an established phenomenon known to occur across the classes Scyphozoa, Hydrozoa, and subphylum Anthozoa (Holstein et al. 2003; Fujita et al. 2021). It extends from minor healing and budding to complete body regeneration; in limited cases, developmental regression from medusa to polyp is possible as well (Piraino et al. 1996). Recent work on newborn ephyrae (juvenile motile life stage of scyphozoans) of *Aurelia* showed a new pathway for injury response where severely damaged ephyrae achieve re-symmetrization and functionality without full replacement of lost appendages (Abrams et al. 2015). A similar response to damage was described in the rhizostome *Mastigias* sp., the sea nettle *Chrysaora pacifica*, and the mediterranean jellyfish *Cotylorhiza tuberculata* (Abrams and Goentoro 2016).

*Cassiopea*, also known as the upside-down jellyfish, is a genus of the class Scyphozoa that lives in symbiosis with dinoflagellates of the family Symbiodiniaceae. It is known to quickly regenerate after damage (within a few days), with work dating back to as early as 1907 establishing adult's capacity to replace severe bell and oral injuries induced in a laboratory setting (Zeleny 1907; Stockard 1908; Ostendarp et al. 2022). *Cassiopea xamachana* medusae may also generate entire new conjoined individuals from damaged bell tissues (Gamero-Mora et al. 2019). Similarly, *Cassiopea* polyps are capable of regeneration (Curtis and Cowden 1974; Newkirk et al. 2022; Mammone et al. 2023) and when cut in half they completely regenerate in 24 days (Newkirk et al. 2022). These regenerative capacities so well established in other life stages of *Cassiopea* (polyps, medusae) have not been fully explored in *Cassiopea* ephyrae (i.e., juvenile jellyfish), and it is not known whether regenerative capability changes with the age of the ephyra. With this paper, we aim to characterize the regeneration capabilities of ephyrae and test whether such regenerative capability decreases during development. In addition, using actin staining, we describe the muscle organization of the *C. xamachana* ephyra, and the reorganization that occurs during symmetrization.

## Materials and methods

### Ephyra care

*Cassiopea xamachana* ephyrae were released from laboratory-grown polyps from a single genotyped colony (GenBank accession number: MZ343250). Strobilation was induced using 40 uM indomethacin (Cabrales-Arellano et al. 2017). From release, the ephyrae were held in individual containers at a mean of 60 µmol m^−^^2^ s^−^^1^ PAR, 24.5 ± 0.5°C, 7.1 ± 0.5 mg/L DO, 35% artificial seawater, with daily water changes and weekly container surface cleaning.

Ephyrae were separated into 10 groups: cut within 24 h of release (group “d1”), cut 3 days post-release (group “d3”), cut 8 days post-release (day 8), cut 15 days post-release (group “d15”), cut 29 days post-release (group “d29”), and control groups for each cut date who underwent photography but not bisection (see Table 1). Ephyrae were dipped into 2% MgCl for <1 min, then staged on agar plates. Experimental ephyrae were cut into two fragments using scalpels to split the bell and manubrium. Size 00 insect pins were used to hold ephyrae in place as they were cut. Regeneration of the two fragments was followed for 15 days until two complete ephyrae developed from the fragments. Photographs were taken for all individuals on the day of release of the ephyra using a Leica microscope and Leica Acquire software v 3.4.1. Ephyrae with failed bisections and/or significantly aberrant morphologies on the day of the cut were removed from the experiment (Table 1). Photographs were taken each day for 1 week, then re-taken 2 weeks post-cut.

**Table 1.** Table summarizing the sample numbers used for the experiment.

Treatment (age of ephyra at cut)	# of ephyrae cut	# of half ephyrae that passed QC	Proportion passing QC	# ephyrae in control
Day 1	10	19	95%	5
Day 3	10	19	95%	5
Day 8	9	16	89%	5
Day 15	10	18	90%	5
Day 29	7	10	71%	5

For every treatment, the number of days since the ephyrae were released from the polyp, the number of ephyrae bisected, the number of halves passing quality control (QC) after the cut, the proportion of ephyra halves passing QC, and the number of ephyrae in the control group are specified.

The angle of tissue wound closure was recorded across the 2 weeks to record symmetry regain. The wound angle starts at ∼180° on cut day, while an angle of zero reflects a closure of the bell rim on the wounded side. Because high manubrium-to-bell ratios have been seen in malformed and starving *Aurelia* and *Cassiopea* (Fu et al. 2014; Muffett et al. 2022), the oral arm-to-bell diameter ratio was recorded on the day of the cut and 2 weeks later to identify morphological abnormalities produced by the recovery process. All ephyrae were provided with eight *Artemia*/day starting 24 h after the bisection.

### Statistical analyses

Time to wound closure across ephyra cut age groups (d1, d3, d8, d15, d29) was non-normal (Kolmogorov-Smirnov one sample test [ks.test], *P* < 0.001), and as such was first compared using a Kruskal-Wallis Test, then a Dunn Test with Holm adjustment (dunn.test). A linear model of cut age groups was built using the r stats lm command, then checked for nonconstant variance, SW normality, and goodness of fit (plot(lm), shapiro.test, car::ncvTest). Closure angles were heteroscedastic and non-normal (Breusch-Pagan [bp.test] and Kolmogorov-Smirnov [ks.test], *P* < 0.001).

Oral arm to bell ratios were computed as average oral arm radius over bell radius. Final oral arm to bell ratios were based on the change in oral arm to bell ratio between precut day and 15 days after cut. This metric was not normal, so was compared between control and cut groups using Wilcoxon tests (wilcox.test).

Likelihood ratio tests using the epitools package (oddsratio) were used to compare survival probability between control and treatment ephyrae. Plots were generated using the “ggplot2” package. All data processing was R v 4.4.2.

### Actin staining

Twelve 5 mm-diameter ephyrae were selected for acting staining. The ephyrae were produced in [the Sea Life Facility at Texas A&M University at Galveston], from polyps originated from settled larvae of mature adults collected from a shallow channel facing Coco Plum Rd S, Key Largo FL. Ephyrae were cut in half (as described above) and kept unfed at a temperature of 25°C, salinity of 33ppt, PAR = 20–50 μmol m^−^^2^ s^−^^1^, and a 12 h light cycle. Halves were stained at *t* = 0 (i.e., immediately after cut), *t* = 24 h, *t* = 48 h, *t* = 72 h post amputation. Staining was performed using a modified protocol from Abrams et al. (2015). More specifically, after anesthetization in MgCl_2_ for 10 min, ephyrae were fixed in 3.7% (vol/vol) formaldehyde for 20 min, washed in PBS, dehydrated and rehydrated in isopropanol series, permeabilized in 0.5% Triton/PBS for 30 min, washed in PBS, and then incubated overnight, in the dark, in 1:20 phalloidin (Alexa Fluor 488 Phalloidin, Life Technologies) solution (in PBS/0.5% Triton). Ephyrae were then washed in PBS, mounted on a slide and imaged using a confocal microscope (ImageXpress Pico, Molecular devices).

## Results

### Ephyrae wound healing

**Fig. 1.**
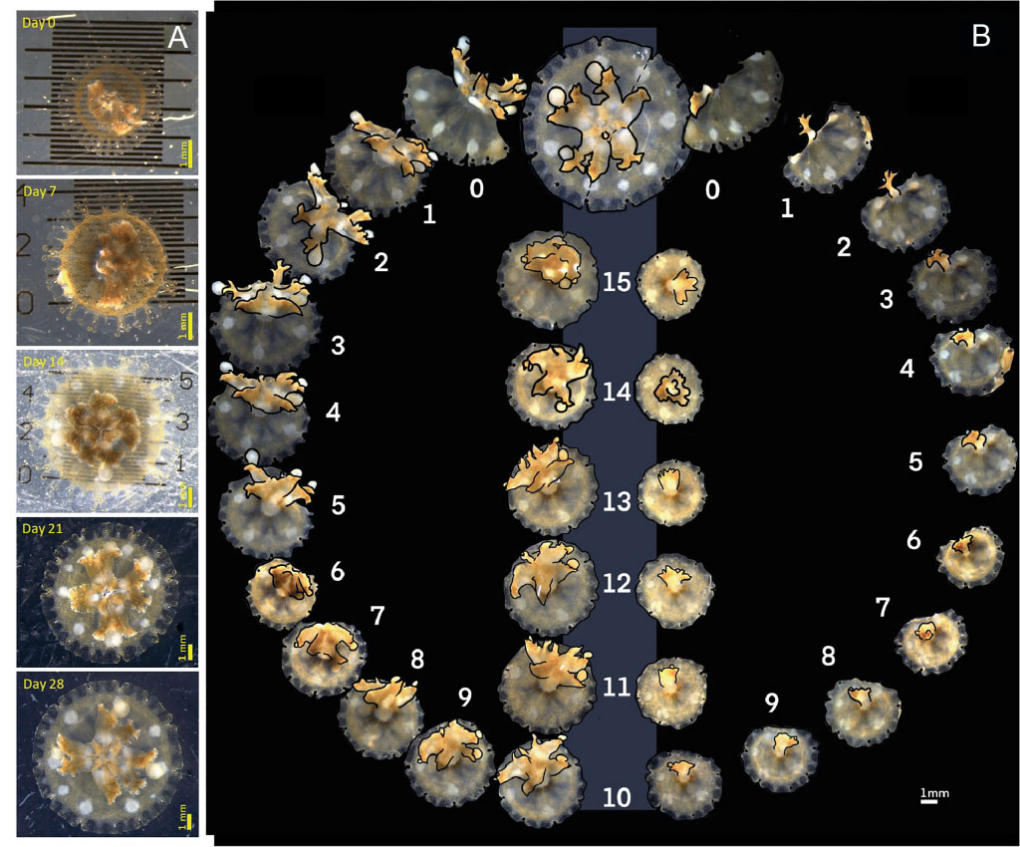
Stereo microscope pictures of *C. xamachana* ephyrae showing growth of intact and bisected ephyrae. (A) Trajectory of growth in the original ephyra with photographs at days 0, 7, 14, 21, and 28. (B) Visual progress of two fragments with circle back comparison to original medusa, all are on the same scale (see scale bar). Locations of rhopalia are pinpointed with black dots, edges of the bell and oral arms are outlined in black. See description of one ephyra regeneration and development in Supplementary Material.

**Fig. 2.**
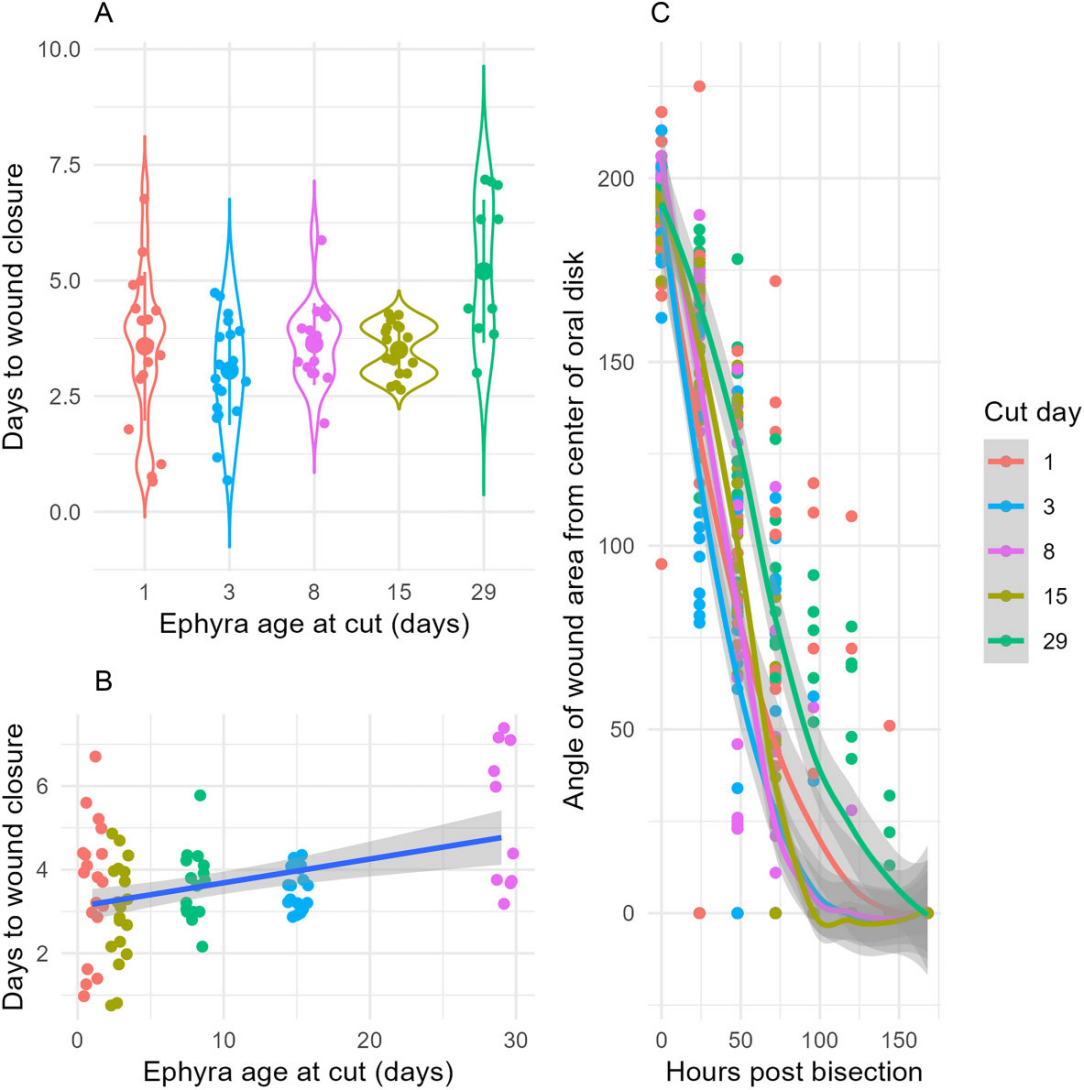
Plots illustrating how ephyrae belonging to different age groups performed regeneration after bisection. (A) Violin plots of the mean (larger circle) ± 1 SD (central line) of the number of days to full wound closure across experimental ages. (B) A linear regression displaying the cut date on the *x*-axis and the number of days the wound took to close on the *y*-axis. (C) Plot of wound angles over time in hours post bisection by treatment groups.

We cut 46 ephyrae in half at different ages to assess their regenerative capability and test whether regenerative potential diminishes with ephyra age. We obtained 92 ephyra halves, of which 82 passed quality control and were kept for downstream analyses (Table 1). Of these, 63 halves generated functional ephyrae that displayed normal morphology and behavior. All ephyrae, regardless of age at cut, closed wounds within 7 days (Fig. 1). Ephyra age had a small positive correlation with number of days to wound closure (ANOVA, *P* = 0.0003; lm Days to heal ∼ Cut date, Estimate: 0.0569 days/day, Adj Rsq: 0.14, F stat: 14.2). Mean time to wound closure was 3.65 days, with few individuals resealing the wound in under 2 days and over 5 days. Mean time to wound closure was 3.58 days for d1 treatment, 3.05 days for d3, 3.62 days for d.8, 3.5 days for d15, and 5.2 days for D. 29 (Kruskal-Wallis test significant and the linear model significant with upward trend, Fig. 2, supplementary). In pairwise testing, time to closure is statistically significantly different between d3 and d15 (*P* = 0.001), and d1 and d29 (*P* = 0.030).

Wound angles across the experiment decreased unsteadily. Within the first 72 h, all groups decreased at a rate of approximately 50°/day. The mean wound angle decreased from 195° (standard deviation ± 15.5) to 146.5 (standard deviation ± 46.5) in the first 24 h, with the subsequent 2 days showing similar declines. A small subset of medusae closed within 1 day (5/82).

### Ephyra regeneration

Abnormalities in bell shape were seen during ephyra development post-cut, but most abnormalities did not persist after the 14th day of healing. The most common of these was loss of planarity, with medusae primarily folding along the bell margin as the wound closed, with tightening along the outermost muscle ring relative to the reset of the bell (Figs. S1, S2). This tightening could be either towards the oral or aboral surface of the medusa but corrected itself within a period of days. Limited areas of imperfect tissue (flatter bell margins) persisted through to 14 days in some individuals.

**Fig. 3.**
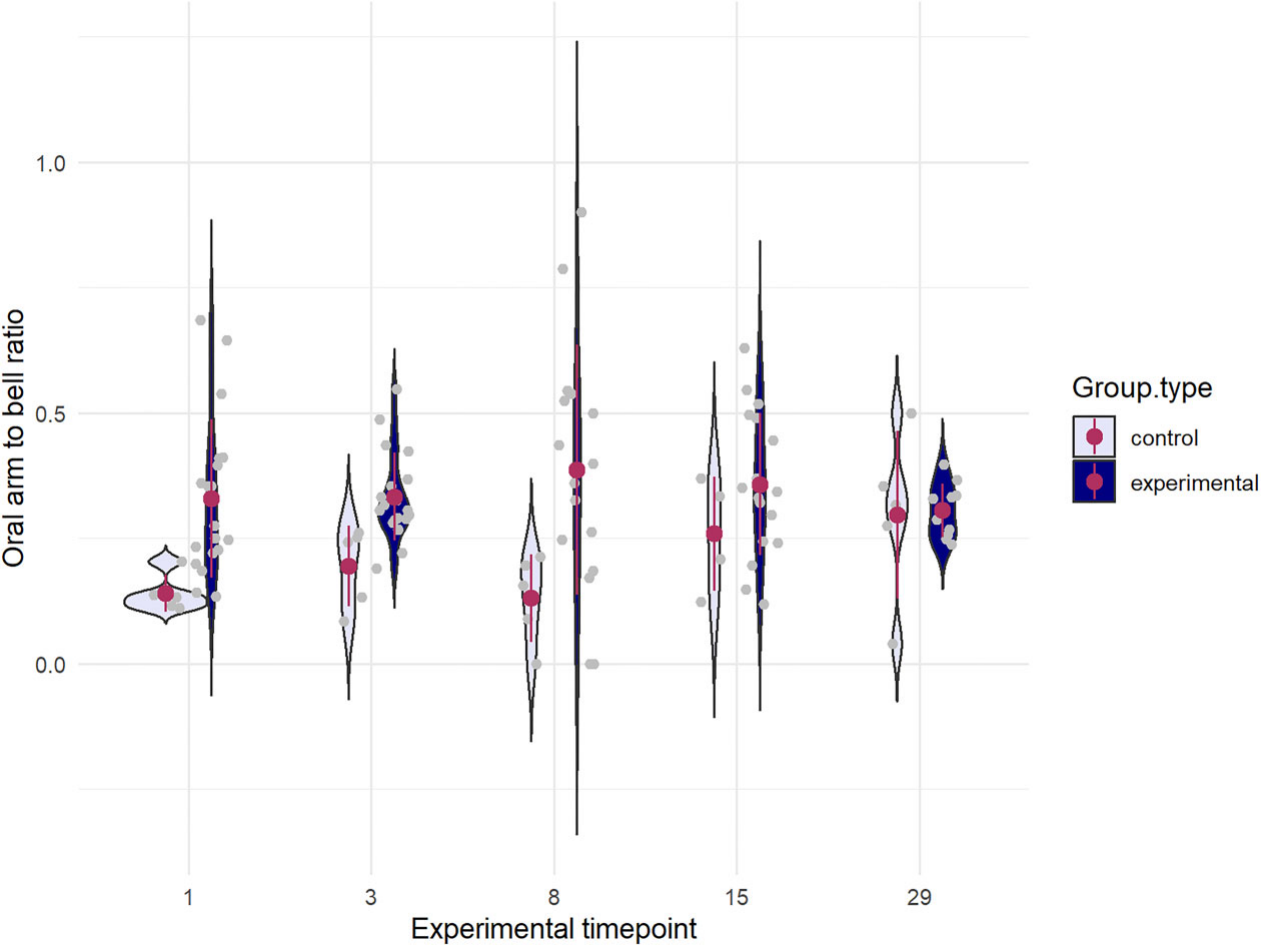
Violin plot illustrating the bell to oral arm ratio of all individuals by cut date at 14 days post injury. Red circle represents mean, line represents ± 1 SD. Higher ratio means more oral arm or manubrium, lower means more bell.

The main change in ephyra form was an increase in oral arm relative to bell radius in cut ephyrae from days 1, 3, and 8 relative to control ephyrae, a change not present in ephyrae cut at day 15 or 28 (Mann-Whitney U, d1: *P* = 0.029, d3: *P* = 0.075, d8: *P* = 0.028, d15: *P* = 0.837, d29: *P* = 0.902; Overall *P* = 0.0007) (Fig. 3). As the original incision halves the digestive cavity, gastric leakage likely persisted for several days.

**Fig. 4.**
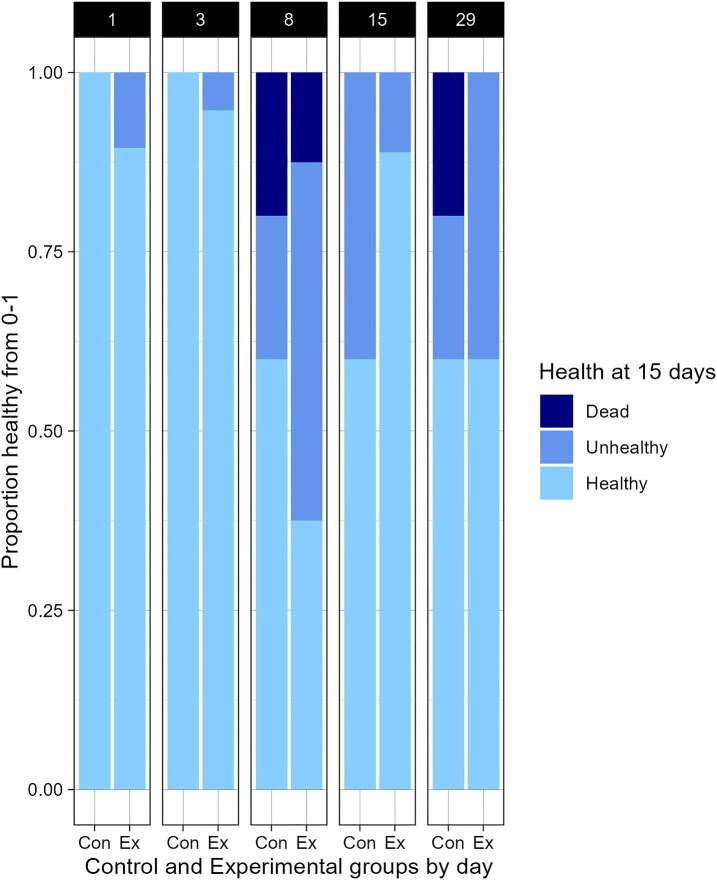
Proportional bar plot containing the proportions of healthy, unhealthy, and dead ephyrae in each treatment and control, 15 days post cut.

At the end of the 14-day trial, ephyrae that ate and pulsed with vigor were deemed healthy. Ephyrae pulsing weakly or failing to eat were deemed unhealthy. No deaths were recorded in Group d1, d3, d15, and d29; 12.5% died in Group d8, 20% died in Control groups d8 and d29 (Fig. 4). Some unhealthy ephyrae were recorded in each experimental group, and in control groups d8, d15, and d29 (see Table S1). However, there was no significantly increased risk of unhealthy development or death between treatment and control (chi sq, *P* = 0.931) (Fig. 4). Ephyrae that did not pass QC (halves that did not include oral arms), *n* = 4 were removed from statistical analyses but were followed—these ephyrae also completed bell repair and by 14 days showed signs of manubrium reformation.

### Musculature of *Cassiopea xamachana* ephyrae

**Fig. 5.**
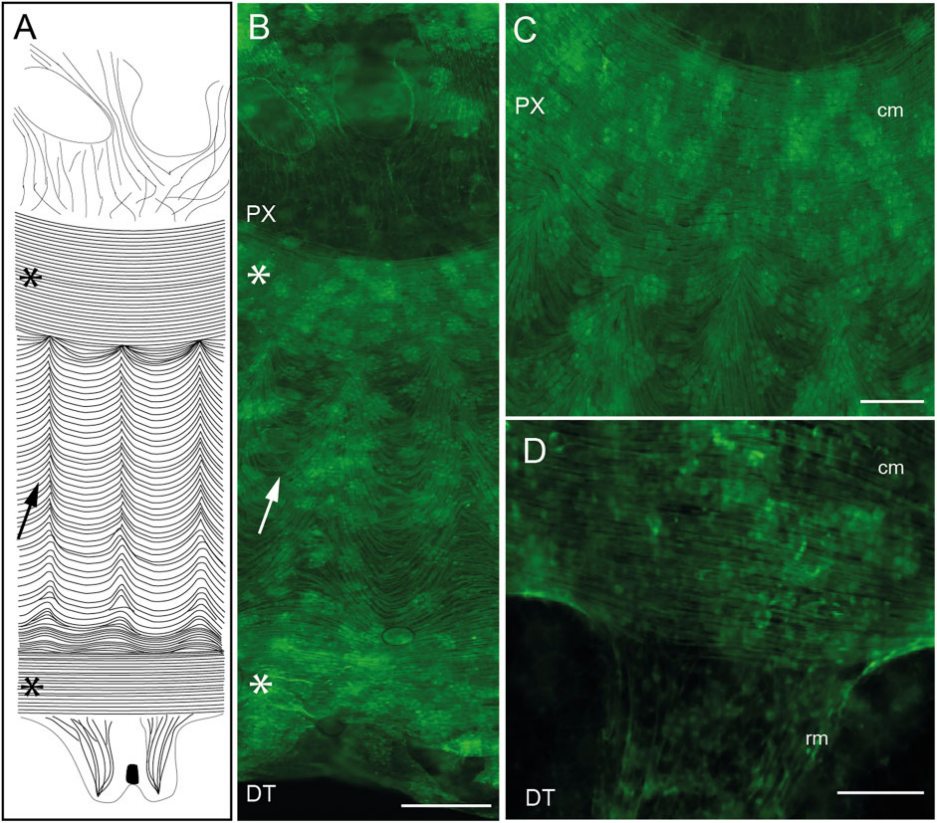
Drawing of the bell musculature of *C. xamachana* and pictures of details of stained ephyrae. Both drawing and pictures follow the same orientation, with the top of each figure facing towards the mouth of the ephyra, and the bottom facing the periphery of the bell. (A) Schematic drawing of a *C. xamachana* ephyra's musculature observed from the oral side. (B) Phalloidin-stained fraction of the oral side of the ephyra, from the base of the oral arms to the rim of the bell. (C) Phalloidin-stained detail of the transition (top to bottom) from the innermost circular coronal band to the undulated coronal band. (D) Phalloidin-stained close up of the intersection (top to bottom) of the outermost coronal muscles with the radial musculature. rm = radial muscles; cm = coronal muscles. PX and DT indicate proximal and distal ends from oral arms. Arrows indicate undulated muscles band, asterisk indicate circular coronal muscles. Scale bar B = 100 µm. Scale bar C, D = 50 µm.

We used phalloidin staining to map the muscle structure of *Cassiopea* ephyrae (Fig. 5). *Cassiopea* ephyrae muscles have mostly been found in the subumbrella. Phalloidin-positive fibers are visible at the base of the oral arms, oriented oro-aborally (Top portion of Fig. 5B). Stained concentric fibers circumvent the base of the oral arms, starting a wide coronal muscle band that extends to the edge of the bell. Within this band the fibers are oriented in two different configurations. Circular tightly organized fibers are found at the inner and outer edges of the muscle band (Fig. 5). In between the two circular layers, the fibers assume an undulated pattern with aligned crenulations (Fig. 5A, B, C). Within the lappets the organization of the stained fibers changes again, organizing centrifugally and forming the radial musculature (Fig. 5A, D).

**Fig. 6.**
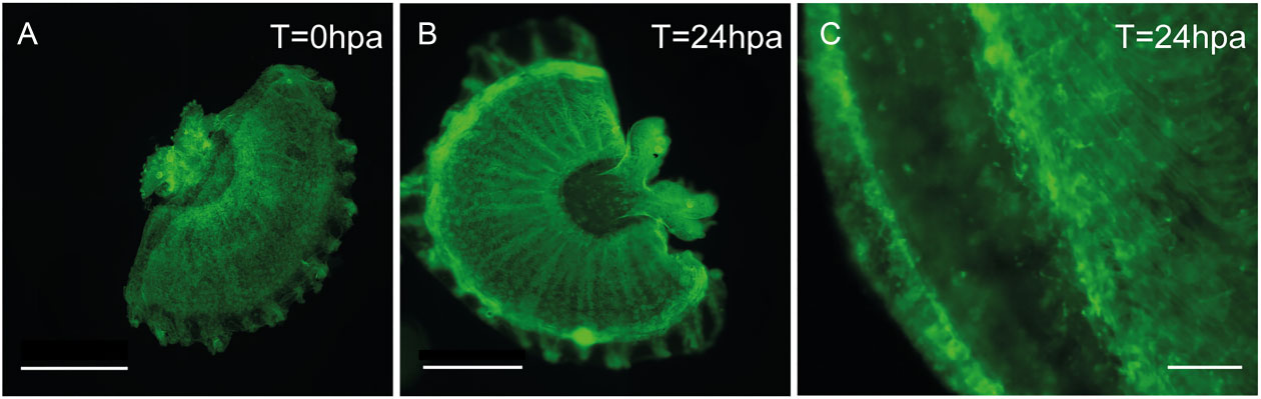
Confocal microscope images of actin-stained regenerating ephyrae at timepoints 0 and 24 h after bisection. (A) *T* (time) = 0 h post amputation (hpa); (B) *T* = 24 hpa after cut; (C) Close up of the wound site at *T* = 24 hpa. Scale bar A, B = 1 mm. Scale bar C = 50 µm. See Fig. S6 for *T* = 48 hpa and *T* = 72 hpa.

In order to identify whether *Cassiopea* bell architecture begins to regenerate musculature early after wounding, we photographed ephyrae immediately after cut (*t* = 0) and at three additional time points after cut (*t* = 24 h, *t* = 48 h, and *t* = 72 h). At 24 h, phalloidin signal was higher in the vicinity of the injury in comparison with the surrounding tissue (see Fig. 6B and white arrow), however by 48 and 72 h, the half-bells became too distorted for analysis of the injured edge.

## Discussion

The hardiness of *Cassiopea* adults and polyps is well documented and multiple publications have reported regeneration of entire body regions in adult medusae and in polyps (Ostendarp et al. 2022; Mammone et al. 2023). *Cassiopea* polyps are constantly reproducing asexually and adult medusae regularly heal from large-scale wounds (Zeleny 1907; Stockard 1908), occasionally including complete regeneration of oral arms or bell. *Cassiopea* medusae can also regenerate from bell fragments, and new medusae can bud from the bell of adult ones (Gamero-Mora et al. 2019; Ostendarp et al. 2022).

In this paper we show that ephyrae, divided into two halves by physical cut, produce complete, and functional duplicate ephyrae within 2 weeks, with wound closure in under a week. Fourteen days after the trauma, the area of damaged tissue was completely sealed, and for many ephyrae the area was visually indistinguishable from the original tissue. Remarkably, these regenerative capabilities remain high across the first month of ephyra life, with half medusae obtained from the cut of newborn, 3-, 8-, 15-, 29-day-old ephyrae forming fully functional individuals in the same amount of time (2–5 days). Although we show no changes in the regenerative capability, we observed a limited impact of the timing of the ephyrae in relation to age. Still, our observed time to recovery is much faster than that observed in adult medusae. Early studies on adult medusae reported a time of recovery after trauma of 29 (Zeleny 1907) to 38 days for umbrellar margin or total oral arm removal (see Stockard 1908), hinting at significant differences in the regenerative timing in ephyra versus adult.

There are many potential explanations for this differentiation in timing; the difference in size between *Cassiopea* ephyra and adult may impact the recovery speed (Zeleny 1907; Stockard 1908; Gamero-Mora et al. 2019). Additionally, tissue layers are thicker, and mesoglea is more abundant in adults, which may impact the timing of regeneration. Future studies should focus on how and when regenerative capability and healing speed change in the developmental trajectory from ephyra to adult medusae.

We used staining with phalloidin to follow muscle fibers in the first hours after cutting. Although we stained jellyfish three times after cutting, we were able to clearly observe the wound site only at *t* = 0 and *t* = 24 h, finding an increased phalloidin signal and inward curvature of the subumbrellar fibers at 24 h (Fig. 6B). A similar staining approach was used to study wound healing in the ephyrae of the genus *Aurelia* (Abrams et al. 2015). Fifteen minutes post amputation, *Aurelia*’s muscles at the wound margins are blunt, and at *t* = 24 h the ends of the coronal muscle extend toward each other. By 48–96 h post amputation, *Aurelia* regained symmetry. The time needed by *Aurelia* ephyrae to regain symmetry is similar to that needed by *Cassiopea*, as is the pattern of wound closure. Unlike *Aurelia*, however, the *Cassiopea* ephyra bell is tightly muscled along a continuous distal edge, resulting in “cup” or “cone” bell warping during bell reconnection. Like *Aurelia, Cassiopea* retains the smaller number of oral arms after symmetrization. *Cassiopea*’s cupping behavior may indicate that displacement-based wound closure occurs in even ephyrae for whom this closure comes with temporarily impaired locomotion but impedes accurate imaging of the medusa. Future studies should examine the impact of this bell distortion that we believe to be a common feature of catastrophic injury recovery in *Cassiopea*. More comparative studies are needed to better understand between species similarities and differences of muscle fiber behavior after injury.

Muscle fibers are located within the basal extension of epitheliomuscular cells, where they aggregate to form a muscle field linked to the mesoglea. Muscular components in cnidarians, particularly in medusae, supply the force for pulsation and motility (Blanquet and Riordan 1981). During the transition from polyp to ephyrae (i.e., strobilation), striated muscles arise *de novo* in ephyrae (Helm et al. 2015) and develop into two striated muscles group: circular coronal muscle and radial muscles as observed in our study (Fig. 5). *Cassiopea* ephyrae morphology of muscles appears to be similar to other species such as *Pelagia noctiluca, Chrysaora quinquecirrha, Chrysaora achlyos*, and *Aurelia aurita* (Helm et al. 2015; Zimmerman et al. 2019). However, there are visible differences in the structure of the coronal musculature. Young ephyrae of the species *Pelagia noctiluca, Aurelia aurita*, and *Chrysaora quinquecirrha* exhibit a circular coronal musculature organized as a continuous ring of fibers running circularly in parallel and interposed between the radial muscles and the mioepythelial musculature surrounding the mouth (Helm et al. 2015; Zimmerman et al. 2019). *Cassiopea xamachana* ephyrae exhibit instead a tripartite circular coronal musculature, with an outer ring of fibers running circularly in parallel and communicating with the radial musculature, an intermediate section with the fibers still running circularly but arranged in a draped pattern, and a smaller inner muscle ring structured like the outermost one (Fig. 5). The mechanical forces exerted by a different subumbrellar musculature could be a possible explanation to why it was common for *Cassiopea* ephyrae in this experiment to regenerate into a cone-like shape, while *Aurelia* ephyrae kept their planarity and gradually distended their coronal muscles (Abrams et al. 2015).

In addition to musculature, we see impacts of regeneration on bell to oral arm ratio. This higher relative oral arm size may be indicative of starvation or food stress during recovery (Fu et al. 2014).

## Conclusions

In this paper, we show that the ephyra of *Cassiopea*, when halved, regenerates two full ephyrae in 3–5 days. Halves from different aged ephyrae (3-, 8-, 15-, and 29-days old at the time of cut) could reconstitute full ephyrae from the halved bodies, with a minimal but significant increase of time needed for regeneration as age progressed. The regenerative capabilities and short time required to rebuild symmetry are remarkable, especially compared to adult *Cassiopea*, which have been shown to heal wounds in significantly longer time (29–36 days). The fact that all ephyrae, regardless of age group regenerated, but took slightly longer time as ephyrae aged, hints at a possible age- or size-dependent effect of healing, which is worth future inquiries. Given the short time required to reconstitute two ephyrae from two halves, regeneration may happen in nature and be of thus some ecological impact in wild medusae. Finally, our map of musculature in ephyra may contribute to comparative works on muscle development and regeneration in different species.

## Supplementary Material

obaf030_Supplemental_Files D1 Supplementary document includes Table S1 and Figs. S1–S6 as well as a careful account of restoration progression for a single medusa.
D2 Supplementary workbook includes the full data collected during the symmetry regain experiment.
D3 Supplementary workbook includes closure angle data from the course of the experiment for R based analyses.
D4 Supplementary sheet includes statistical comparisons between groups.

## Data Availability

The partial COI sequence for the colony is accessible via GenBank accession number MZ343250. All photographs are available in the associated figshare (https://doi.org/10.6084/m9.figshare.28297349.v1). R code is accessible on the corresponding author's github (https://github.com/kadenmuffett/Cassiopea_symmetry).
