# Supplementary material for: Regeneration and Musculature in Halved Cassiopea xamachana Ephyrae
Source: Integr Org Biol. 2025 Jul 26;7(1):obaf030. doi: 10.1093/iob/obaf030 (PMC12344487; doi:10.1093/iob/obaf030)
Supplement: obaf030_Supplemental_Files — D1 Supplementary document includes Table S1 and Figs. S1–S6 as well as a careful account of restoration progression for a single medusa. D2 Supplementary workbook includes the full data collected during the symmetry regain experiment. D3 Supplementary workbook includes closure angle data from the course of the experiment for R based analyses. D4 Supplementary sheet includes statistical comparisons between groups. [file obaf030_supplemental_files.zip › D1_Supplementary_info.docx]

**Supplementary Material D1**

**Table S1**. Table recording the total number of individuals removed in experimental treatments (E) and control (C), due to a poor incision and a misshapen starting ephyra. Table also shows number of ephyra that were unhealthy or dead 14 days after bisection.

| **Group** | **Total Removed** | **Removed- due to cut error** | **Removed-**  **misshapen ephyra at time of cut** | | **Passed QC, was unhealthy at end of experiment** | | **Passed QC, was dead at end of experiment** |
| --- | --- | --- | --- | --- | --- | --- | --- |
| Day 1 E | 1 | 1 | 0 | 2/19 | | 0/19 | |
| Day 1 C | 0 | NA | 0 | 0/5 | | 0/5 | |
| Day 3 E | 1 | 1 | 0 | 1/19 | | 0/19 | |
| Day 3 C | 0 | NA | 0 | 0/5 | | 0/5 | |
| Day 8 E | 2 | 2 | 0 | 8/16 | | 2/16 | |
| Day 8 C | 0 | NA | 0 | 1/5 | | 1/5 | |
| Day 15 E | 2 | 0 | 2 | 2/18 | | 0/18 | |
| Day 15 C | 0 | NA | 0 | 2/5 | | 0/5 | |
| Day 29 E | 4 | 0 | 4 | 4/10 | | 0/10 | |
| Day 29 C | 0 | NA | 0 | 1/5 | | 1/5 | |
| **Total** | **10** | **C: 0;E: 4** | **C: 0; E: 6** | **C: 16.7%; E: 23.4%** | | **C: 8%; E: 2.5%** | |

**Supplement A: Step by step description of the regeneration and development of one ephyra after halving.**

One 30-day old ephyra was tracked carefully for two weeks after bisection. The ephyra was released from the colony at a size of 3.8 mm in diameter, twenty-four hours before injury (day 29), the ephyra was 9.9 mm in diameter and had 13 rhopalia (see Figure 1). On day 30, the ephyra was sheared in half. The tear split the bell into nearly equivalent fragments (six rhopalia on Fragment A and seven rhopalia on Fragment B). Fragment A retained three oral arms of the medusa’s original four-arm trunks, leaving Fragment B with one damaged arm and a negligible portion of the central gastrovascular cavity (GVC). Immediately after the injury, the medusa halves both pulsed rapidly for five minutes. After these five minutes, the smaller half (hereafter referred to as Fragment B) seized and ceased moving, while the pulses of Fragment A pulsed stochastically for the following fifteen minutes.

By 24 hours after the bisection (day 31), fragment A and B had contracted significantly. Fragment A’s largest diameter was 8.6mm, and Fragment B’s was 8.4 mm. Both fragments had a layer of tissue sealing over the full extent of the tear, along with tissue sealing the minor tears along the bell margins. While the bell restoration in Fragment A was not quantifiable by day because of oral arm positioning (see Figure 2), Fragment B’s bell radius at the center point of the cleaved side increased by an average of 0.16 mm/day +/- 0.11mm for the first ten days post-injury. Bell radius on the damaged side leveled out at ten days post-trauma with a radius roughly equivalent to the intact side (2.56 mm vs. 2.38 mm).

The original medusa had 13 rhopalia. After the trauma, the two fragments had six (Fragment A) and seven rhopalia (Fragment B) each. By 15 days into the recovery process, Fragment A had eight rhopalia, and Fragment B had seven. In Fragment A, the additional rhopalia were the last feature to regenerate in the half-month recovery period. During the recovery, their absence was noticeable in the distinctive curling (Fig S1, S2) of the recovering portion of the bell margin lacking rhopalia and the lethargy of this bell section compared to the surrounding tissue. Fragment A generated rhopalia in a new bell region and in the same cleft as an extant rhopalium. This can be seen in Figure S3, where two rhopalia are nestled in the same cleft between interrhopaliar regions. Similar duplicate rhopalia were observed in other undamaged ephyrae from this same population, but never in newly released ephyrae.

Bell margin complexity grew quickly from the simple straight line of new tissue growth visible 24-hours after cleavage. While radius increase on the damaged portion stagnated ~10 days after the tear (day 40), the complexity of both Fragment A & B’s replacement tissue on the bell margin continued to develop from day 40 through 45. The visibly flattened edges on these sides, with little to no variation between interrhopaliar regions gained the characteristic peak and valley pattern of an undamaged ephyra.

Feeding

Fragments A and B were supplied with eight 20-hr *Artemia* each day following the separation, consistent with the feeding routine of the original ephyra. For the first three days post-separation, both consumed *Artemia* but were unable to digest them, leaving the fully or mostly undigested shrimp in their container. Six days-post separation Fragments A & B possessed signs of having digested their own oral arm tissue (note in Figure 1 the change in color of the bell to a brighter orange and the reduction in oral arm size and complexity). However, oral arm complexity and shrimp consumption gradually increased after this nadir (Fig 1&S4). Fragment B still consumed no more than one brine shrimp per day for the remainder of observation, occasionally exhibiting the gaping behavior characteristic of underfed medusae (visible in Figure S5). Despite this, on days 44 and 45, Fragment B displayed growing oral trunk separation into three distinct oral arms. Fragment A consumed between four and seven Artemia/day.

Pulsation pattern

After the first 24 hours, Fragments A and B took up pulsation in line with other healthy ephyrae (~20-40 ppm) patterns. However, during the first 12 days, their movements remained very limited. Swimming in the water column was not recorded in either fragment until day 45 (last day of observation). Excessive mucus was present for the first week across the bottom of the holding tank.


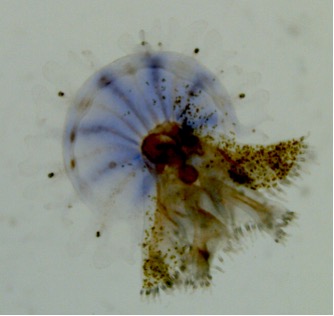


**Supplementary Figure 1**. Bell folding in symmetrized subject c11, four days post bisection.


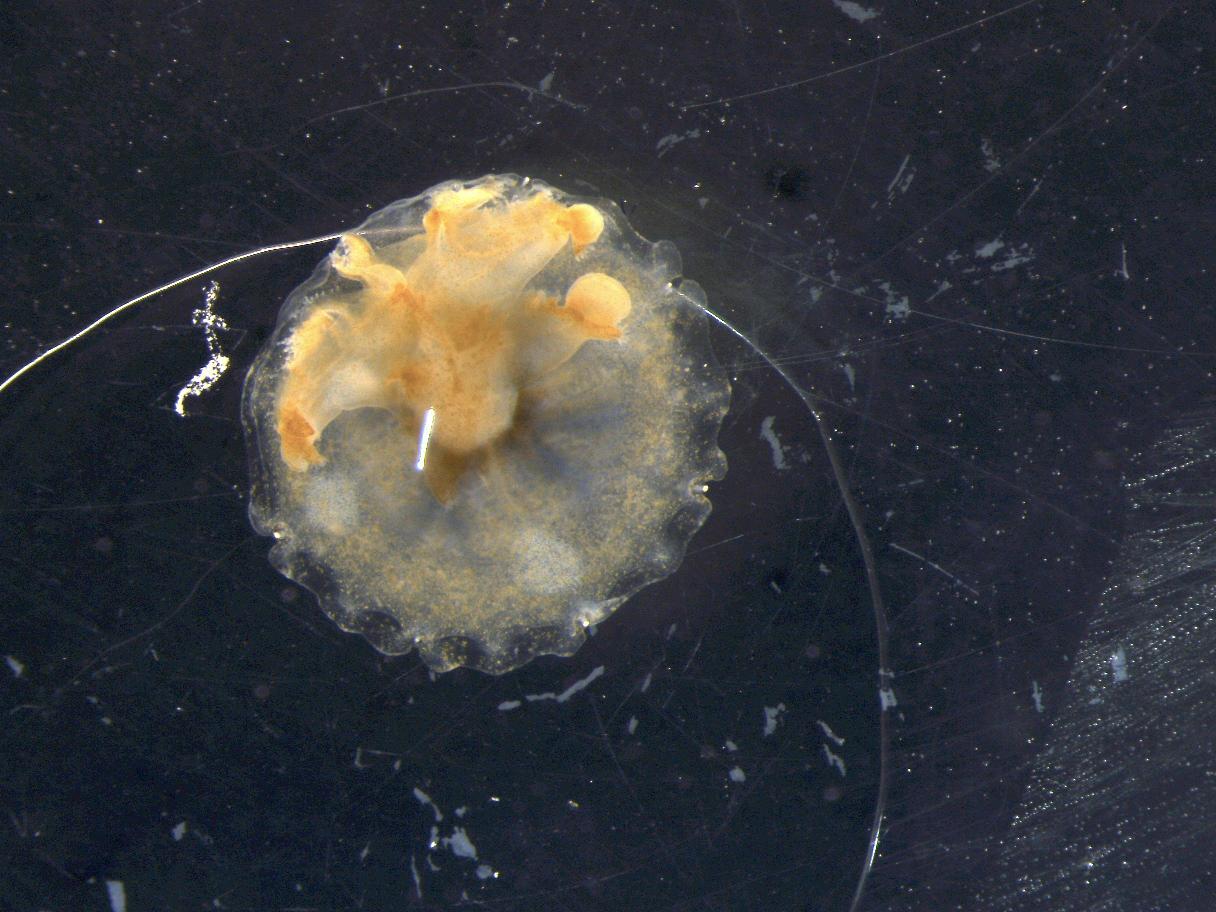

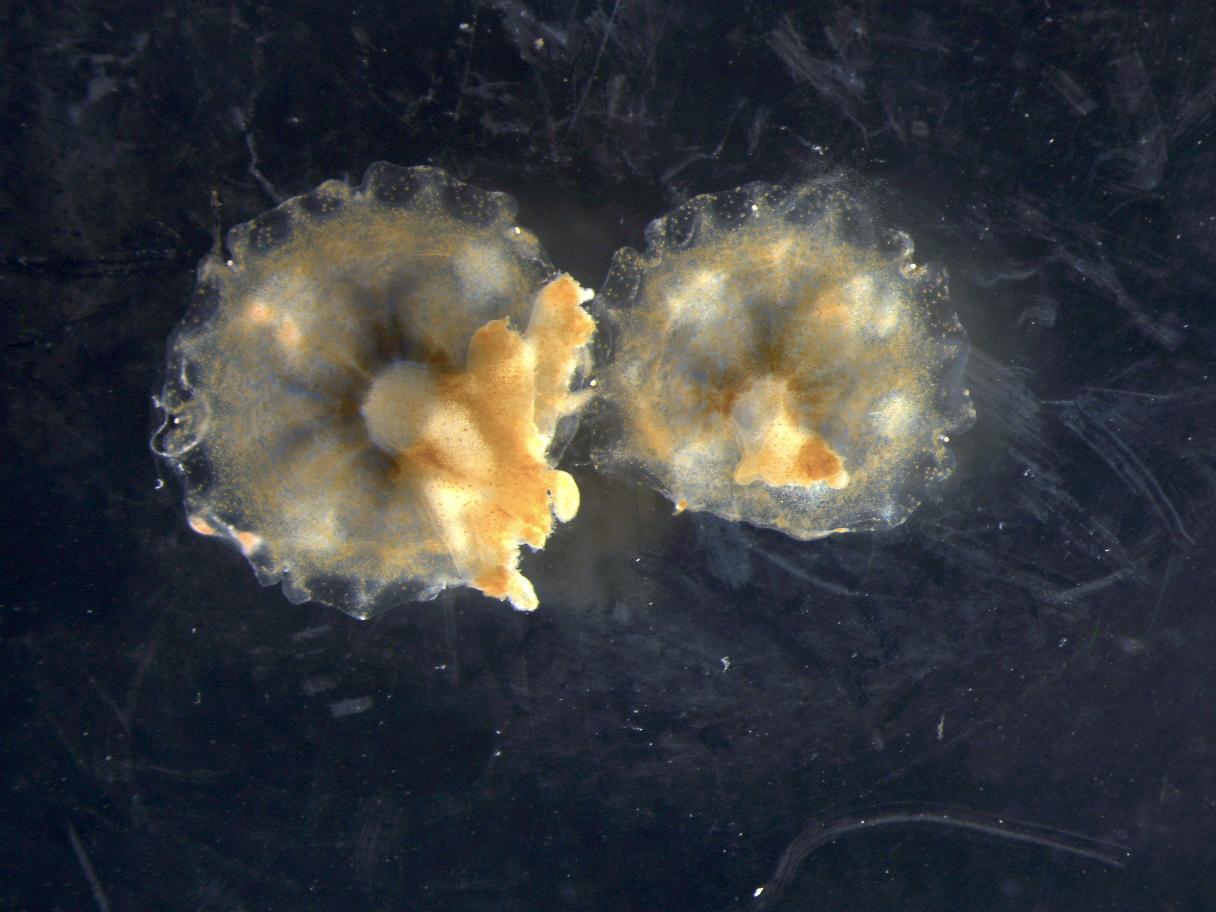


**Supplementary Figure 2**: Bell curling in Fragment A (day 42) and Fragment B (day 38).


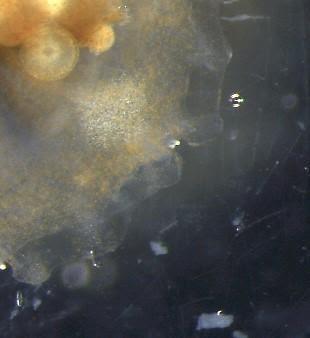


**Supplementary Figure 3**: Duplicate rhopalium/statocyst in Fragment A (day 45).


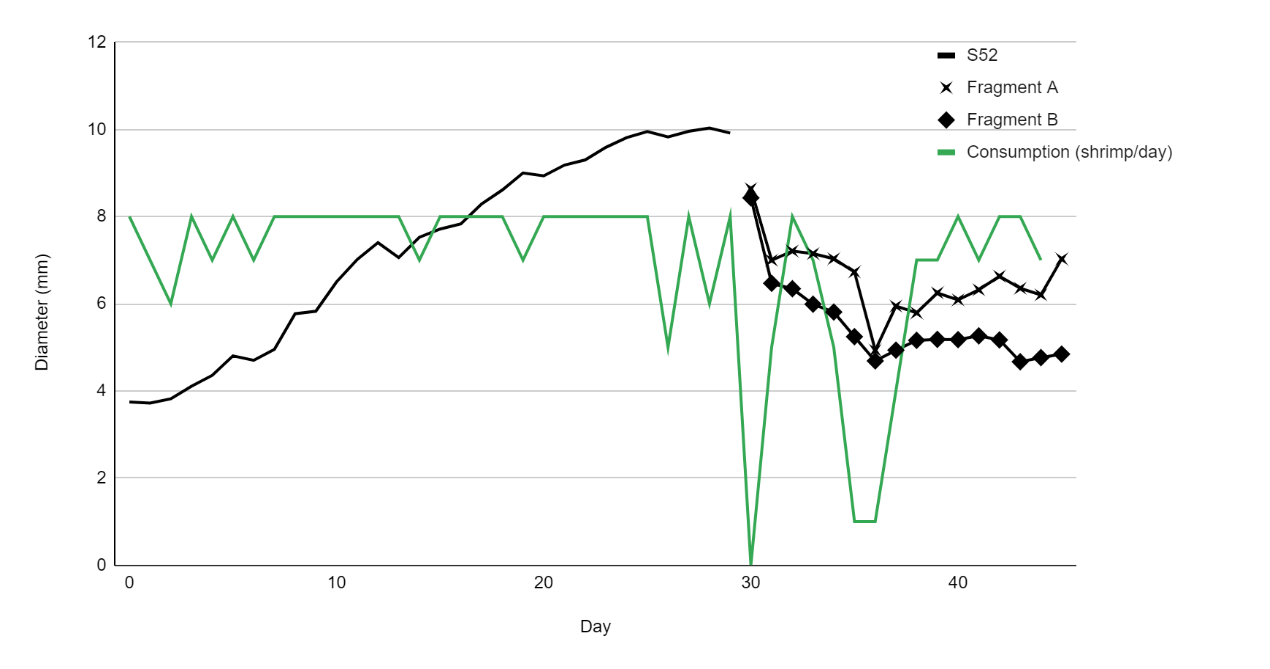


**Supplementary Figure 4**: Trajectory of growth through bell diameter and number of brine shrimp consumed in each 24 period by Fragment A and B together


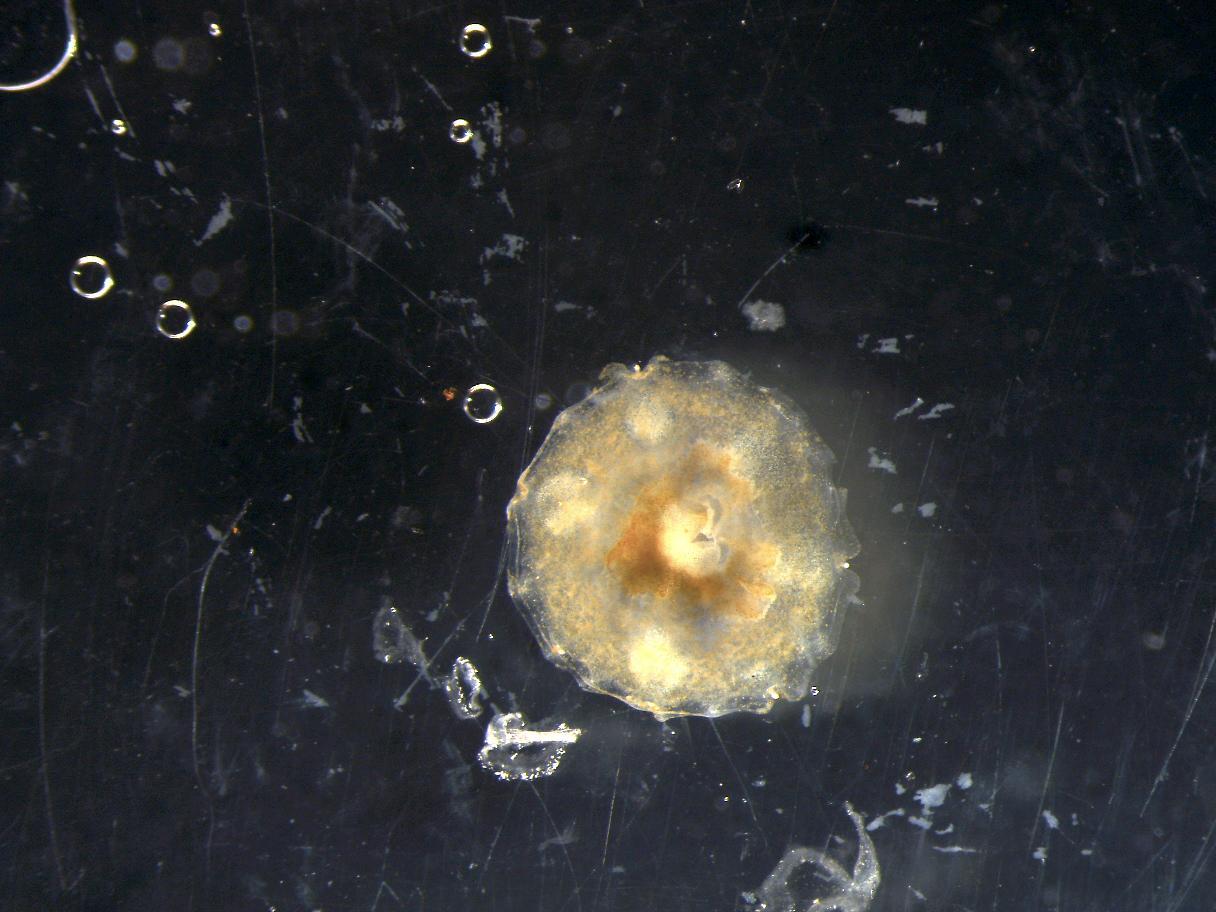


**Supplementary Figure 5**: Fragment B gaping on day 44, note the very visible ingress path into the GVC.


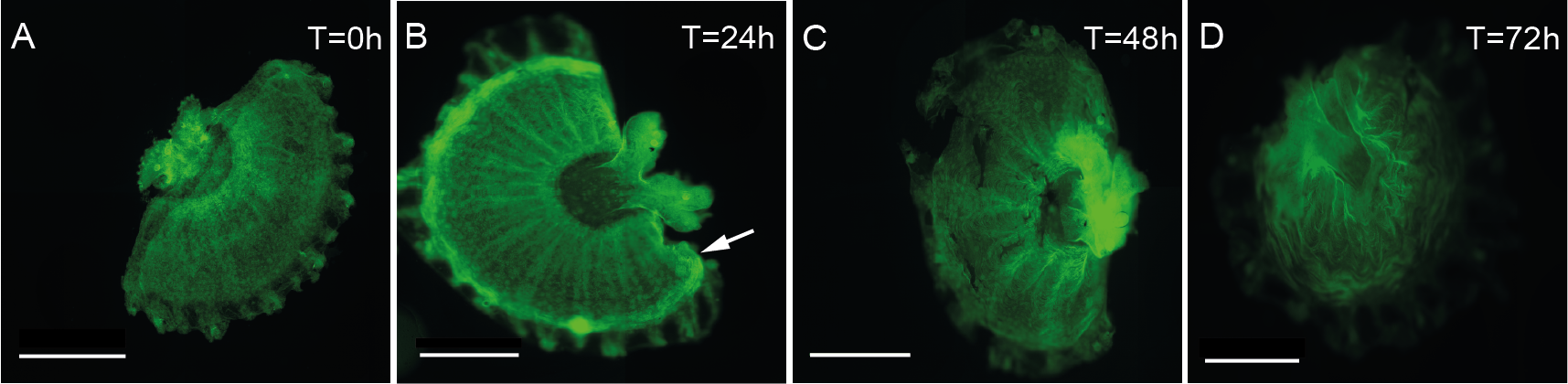


**Supplementary Figure 6**: Confocal microscope images pictures of actin(phalloidin)-stained regenerating ephyrae at timepoints T = 48hpa and T = 72hpa.
